# The exchanged EF-hands in calmodulin and troponin C chimeras impair the Ca^2+^-induced hydrophobicity and alter the interaction with Orai1: a spectroscopic, thermodynamic and kinetic study

**DOI:** 10.1186/s12858-015-0036-7

**Published:** 2015-02-15

**Authors:** Drake Jensen, Nicole Reynolds, Ya-Ping Yang, Shubha Shakya, Zhi-Qiang Wang, Dennis J Stuehr, Chin-Chuan Wei

**Affiliations:** Department of Chemistry, Southern Illinois University Edwardsville, Edwardsville, IL 62026 USA; Department of Chemistry, Kent State University at Tuscarawas, New Philadelphia, OH 44663 USA; Department of Pathobiology, The Lerner Research Institute, Cleveland Clinic Foundation, Cleveland, OH 44195 USA

**Keywords:** EF-hand, Calcium binding, Calmodulin, Troponin C, Orai, ANS, Isothermal titration calorimetry, Fluorescence, Kinetics

## Abstract

**Background:**

Calmodulin (CaM) plays an important role in Ca^2+^-dependent signal transduction. Ca^2+^ binding to CaM triggers a conformational change, forming a hydrophobic patch that is important for target protein recognition. CaM regulates a Ca^2+^-dependent inactivation process in store-operated Ca^2+^ entry, by interacting Orai1. To understand the relationship between Ca^2+^-induced hydrophobicity and CaM/Orai interaction, chimera proteins constructed by exchanging EF-hands of CaM with those of Troponin C (TnC) are used as an informative probe to better understand the functionality of each EF-hand.

**Results:**

ANS was used to assess the context of the induced hydrophobic surface on CaM and chimeras upon Ca^2+^ binding. The exchanged EF-hands from TnC to CaM resulted in reduced hydrophobicity compared with wild-type CaM. ANS lifetime measurements indicated that there are two types of ANS molecules with rather distinct fluorescence lifetimes, each specifically corresponding to one lobe of CaM or chimeras. Thermodynamic studies indicated the interaction between CaM and a 24-residue peptide corresponding to the CaM-binding domain of Orail1 (Orai-CMBD) is a 1:2 CaM/Orai-CMBD binding, in which each peptide binding yields a similar enthalpy change (ΔH = −5.02 ± 0.13 kcal/mol) and binding affinity (K_a_ = 8.92 ± 1.03 × 10^5^ M^−1^). With the exchanged EF1 and EF2, the resulting chimeras noted as CaM(1TnC) and CaM(2TnC), displayed a two sequential binding mode with a one-order weaker binding affinity and lower ΔH than that of CaM, while CaM(3TnC) and CaM(4TnC) had similar binding thermodynamics as CaM. The dissociation rate constant for CaM/Orai-CMBD was determined to be 1.41 ± 0.08 s^−1^ by rapid kinetics. Stern-Volmer plots of Orai-CMBD Trp76 indicated that the residue is located in a very hydrophobic environment but becomes more solvent accessible when EF1 and EF2 were exchanged.

**Conclusions:**

Using ANS dye to assess induced hydrophobicity showed that exchanging EFs for all Ca^2+^-bound chimeras impaired ANS fluorescence and/or binding affinity, consistent with general concepts about the inadequacy of hydrophobic exposure for chimeras. However, such ANS responses exhibited no correlation with the ability to interact with Orai-CMBD. Here, the model of 1:2 binding stoichiometry of CaM/Orai-CMBD established in solution supports the already published crystal structure.

## Background

Calmodulin (CaM) is a small, acidic protein with 148 amino acids, which plays important roles in Ca^2+^-dependent signal transduction in eukaryotes. There are a number of CaM target molecules that have been identified, including, to name a few, protein kinase, protein phosphatase, nitric oxide synthase, tRNA, Ca^2+^ pump, and proteins involved in motility and T-cell activation [1]. CaM is a Ca^2+^ sensor protein in non-muscle cells, which binds four Ca^2+^ ions through its self-contained four Ca^2+^ binding helix-loop-helix structures called EF-hands (EFs). The structure of CaM is arranged into two separated globular lobes, each containing a tandem pair of EFs, with a flexible tether in between. Ca^2+^-free CaM adopts a so-called closed structure, in which the two lobes come in close proximity of each other by burying most of their hydrophobic residues. Ca^2+^ binding to CaM triggers a major conformational change to form an extended dumbbell-shaped structure, linked by a solvent-exposed, rigid helical structure in x-ray crystallography [2-4] but an un-structural linker in NMR [5], suggesting both structures may coexist in solution to facilitate target complexation.

The mechanism for CaM to recognize its target molecules is primarily through strong hydrophobic interactions, in which Ca^2+^ binding to CaM exposes its hydrophobic patch, allowing CaM to interact with the CaM-binding domain (CMBD) of a target molecule followed by enzyme activation. A CMBD typically is comprised of 15–35 amino acids with high propensity for helix formation, which shows an un-structural conformation in solution but forms an α helix when complexed with CaM. The CMBD sequences are considerably divergent. There are several structures of CaM/CMBD complexes that have been determined, including those that fall under the category of the well-documented canonical model. In this model, each lobe of CaM interacts with the different ends of a CMBD peptide in a sequential manner; first binding to the C-terminal lobe followed by the N-terminal lobe [6,7]. To achieve this, CaM’s helix linker is disrupted and extended, forcing the structure to “collapse” to grip the peptide [8]. Despite the overall structural change, NMR reveals that there is no significant conformational change within each lobe between the uncomplexed and complexed states [9]. Classic examples of this canonical binding include CaM/M13 and CaM/CaMKII. Alternatively, other CaM/CMBD complexes have been observed in a non-canonical manner, in which CaM binds target CMBDs with a 1:1, 1:2 or 2:2 mode (for review, see ref. [10]). Despite that the current understanding for recognition allows one to predict the possible CMBD sequences in target enzymes or proteins assisted with the CaM target database [11], the mode of CaM/CMBD recognition still remains to be elucidated only by experimental approaches, indicating that the hydrodynamic properties of CaM and its associated physiochemical properties are not well understood. Therefore, there is still a need to understand the structural basis of the interaction between CaM and its target proteins. An advance in this knowledge will eventually lead to a better understanding of CaM’s diverse regulatory functions.

**Figure 1:**
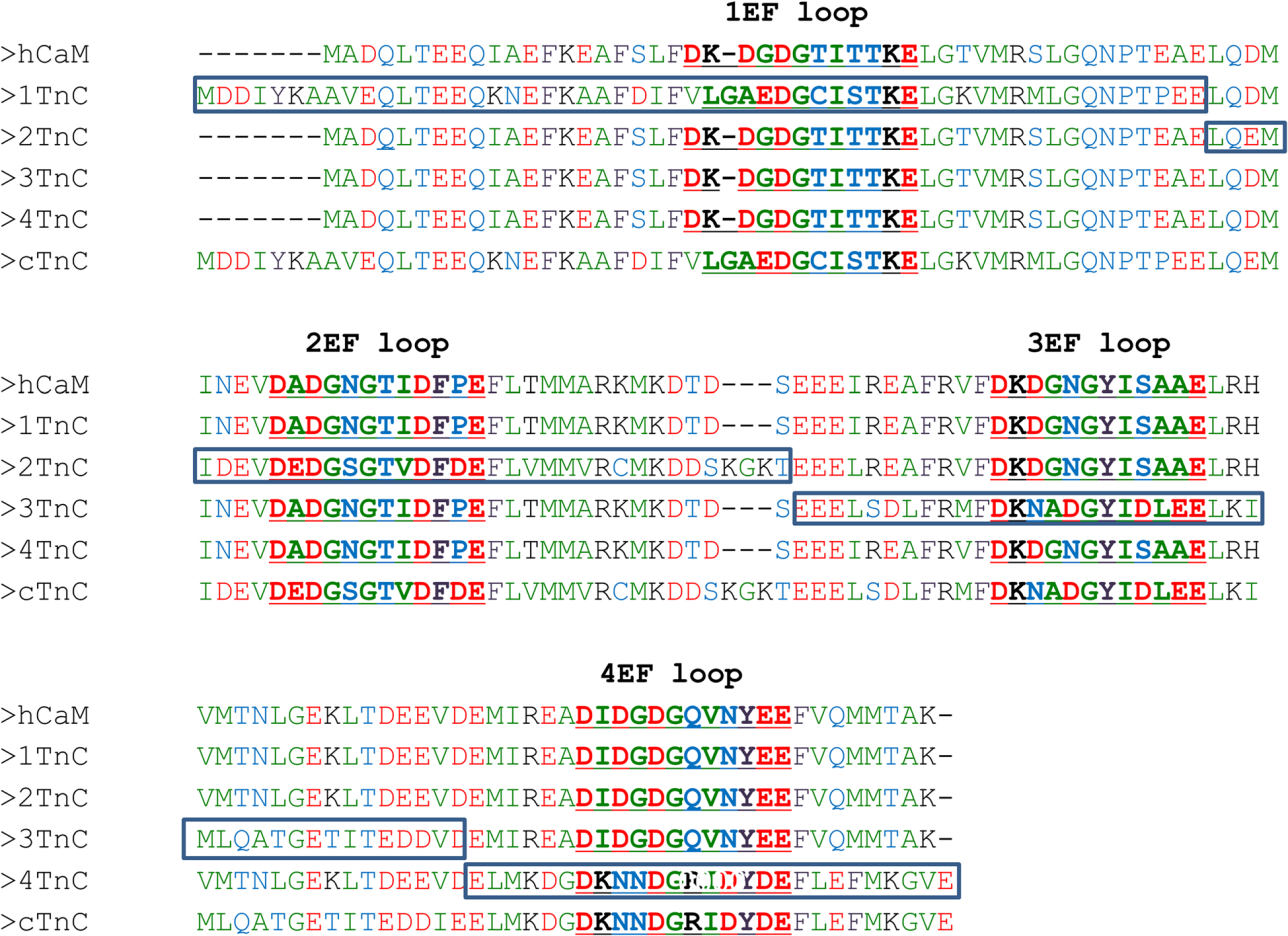
**Sequence alignment of hCaM, cTnC and chimeras.** Sequence alignment was performed with ClustalW. Residues are colored coded in terms of amino acid properties where green are hydrophobic, blue are polar/uncharged, black are positively charged, red are negatively charged and brown are aromatic. The four, 12-residue EF-hand Ca^2+^ binding loops are depicted in bold and underlined. The corresponding sequences from cTnC added to create the individual chimeras are outlined with boxes.

In the past, chimeras of CaM and Troponin C (TnC) have been utilized to help elucidate the binding mechanism to their target proteins. CaM and TnC share only 50–70% homology at the amino acid level but they possess striking structural similarities, as shown in the crystal structures of CaM and skeletal TnC (sTnC). Both CaM and TnC contain four EFs but have an opposite effect in the activation of target enzymes. For example, TnC can neither bind to nor activate nNOS [12] and has a very low affinity for Ca^2+^-ATPase [13]. Given the structural similarities and functional differences of CaM and TnC, chimera proteins, in which the domains containing EFs of CaM are exchanged with the corresponding EFs of TnC, and vice versa, allow for the investigation of the functionality of each specific protein. For example, chimeras of CaM, where each includes one of four domains (either the 1^st^ EF-hand (EF1), EF2, EF3 or EF4) from TnC have been constructed, including CaM(1TnC) (domain 1 of TnC and domains 2, 3, and 4 of CaM), CaM(2TnC), CaM(3TnC), and CaM(4TnC), as well as other multiple-domain exchange chimeras. These chimeras have been used in the studies of CaM/NOS [12,14,15] and CaM/Ca^2+^-ATPase interactions [13]. It is assumed that these chimeras adopt a similar structure as CaM, and therefore interact with CMBDs similarly. Thus, the change of functionality, such as enzyme activation, can be explained mainly by the lack of the association of specific elements in the chimeras (due to the subtle sequence differences and/or side chain packing) and specific domains in the target macromolecules. In some cases, the failure of stimulating enzymatic activation is explained by the lack of complex formation due to the exchanged domains [16]. However, whether or not such exchanged EFs have an impact on chimera protein structure, including hydrophobic exposure, as well as CMBD interactions, has yet to be addressed. Because the Ca^2+^-induced hydrophobicity of CaM is generally believed to be important for its ability to recognize CMBDs, here, through spectroscopy and calorimetry, we first utilized the ANS fluorescent probe to assess the extent of hydrophobicity and structural change of the four chimera proteins upon Ca^2+^ binding. The sequences of the constructs from human CaM (hCaM) and cardiac TnC (cTnC) are summarized in Figure 1. Notable sequence differences among the chimeras include 1) CaM(1TnC) contains additional N-terminal residues and its EF1 is non-canonical in that it does not bind Ca^2+^, and 2) CaM(2TnC) has three extra residues in its central helix. Besides the sequence difference, TnC also has different Ca^2+^ binding properties. TnC’s EF4 has a one order higher Ca^2+^ affinity than that of CaM, and its second helix of EF2 (i.e. helix D) is oriented differently compared to that of CaM.

Our laboratory has long interests in the role of STIM and Orai in store-operated Ca^2+^ entry (SOCE). STIM is an ER-membrane bound Ca^2+^ sensor protein within the ER lumen, which forms as a monomer and oligomer in high and low Ca^2+^ concentrations, respectively. When Ca^2+^ is depleted in the ER lumen, a transient structure from the STIM aggregate formed near the plasma membrane surface activates the Orai Ca^2+^ channel protein, leading to Ca^2+^ influx [17,18]. The Ca^2+^ influx is immediately inhibited by a Ca^2+^-dependent inactivation (CDI) process, mainly through the interaction of CaM and the CMBD of Orai [19]. Here, we studied the interaction of CaM with a peptide corresponding to the CMBD of Orai1 (Orai-CMBD) and further extended the studies to the chimera proteins themselves. Given the fundamental differences between CaM and TnC, we anticipate that these chimeras will serve as an informative probe to better understand the interaction between CaM and Orai-CMBD and therefore, could be used to extend to studies using the whole length of the Orai channel protein as well as to other CaM/CMBD systems. During our characterization, an excellent work published by Liu *et al*. showed that CaM binds to Orai’s CMBD in an unusual 1:2 open conformation mode [20]. Thus, we rationalized our experimental data to accommodate their model.

## Results and discussion

In physiological conditions, the bulk concentration of Ca^2+^ within a cell is kept very low (~100 nM). When the cell is stimulated, its Ca^2+^ concentration is elevated to approximately 1 μM. While this elevated bulk concentration is sometimes insufficient to activate specific signaling processes, the formation of local hot-spots of Ca^2+^, aka Ca^2+^ concentration microdomains (CCMs), has been documented and its concentration can reach tens of micromolar [21]. In our model study, we have used 2 mM Ca^2+^ in all experiments to ensure that CaM and chimera proteins are saturated with Ca^2+^ for a fair comparison.

### Structural change of CaM and chimeras probed with ANS

**Figure 2:**
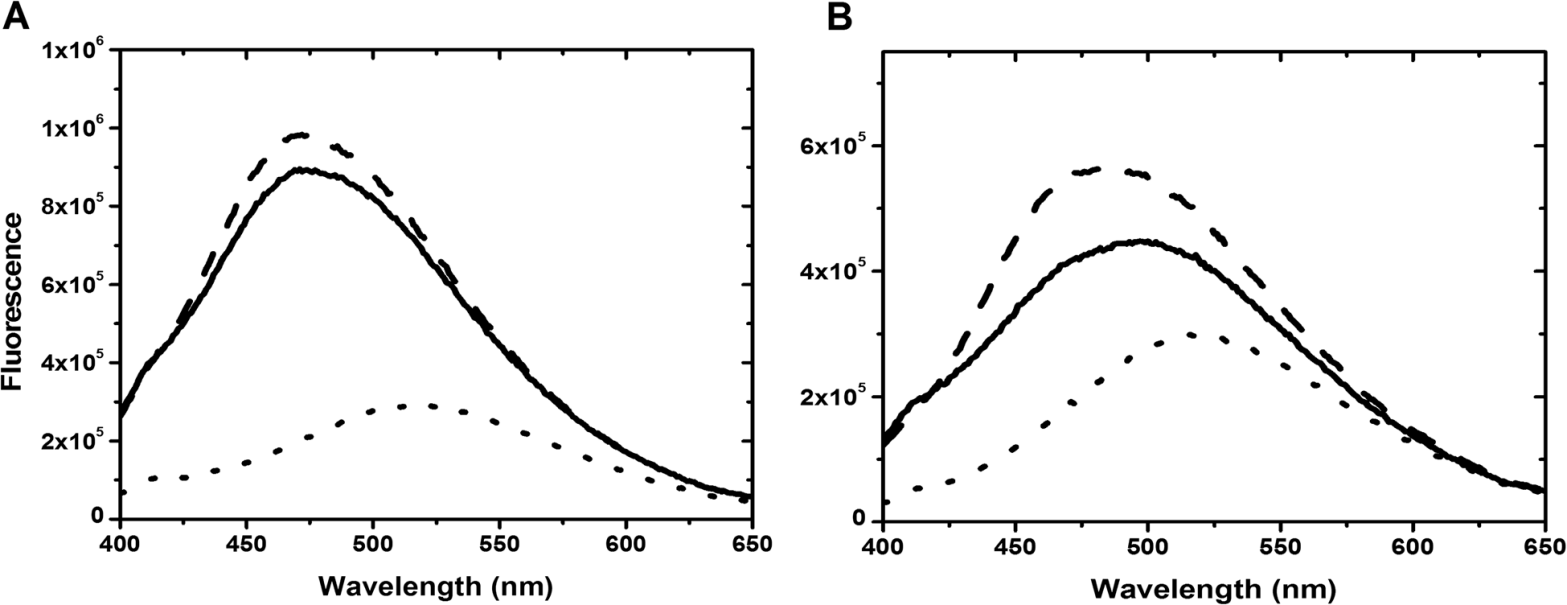
**ANS fluorescence of uncomplexed and complexed CaM and 3TnC. (A)** shows the ANS fluorescence of a solution containing 200 μM ANS and 5 μM CaM in the absence (dotted line) and presence (solid line) of Ca^2+^. The ANS fluorescence of CaM/Orai-CMBD was recorded by adding a solution of Orai peptide into Ca^2+^-CaM until no more intensity change was observed (dashed line). **(B)** shows the ANS fluorescence of CaM(3TnC) in the identical experimental condition as in **(A)**. ANS fluorescence was excited at 350 nm.

It is well known that the exposure of the hydrophobic patches of CaM is essential for target protein recognition. Such hydrophobic exposure can be photometrically monitored by its interaction with extrinsic 1-anilinonaphthalene 8-sulfonate (ANS) and 2-p-toluidinyl-naphthalene-6-sulfonate (TNS) molecules. These two fluorophores have been widely used as a probe for measuring hydrophobicity. ANS is a fluorescent dye which emits very weak fluorescence in water due to its excited charge transfer (CT) state that is quenched easily by water molecules. ANS alone exhibits very little fluorescence with a maximal wavelength (λ_max_) of 520 nm (Figure 2). ANS fluorescence increases dramatically in a hydrophobic environment or when the rotational motion of its phenylamino group is restricted [22]. In the absence of Ca^2+^, the ANS fluorescence of CaM or chimeras exhibited no change, mainly due to the lack of hydrophobic surface and the presence of electrostatic repulsion between the negatively charged sulfonate group of ANS and the negatively charged residues in CaM and chimeras. The fluorescence increased when Ca^2+^ was included in the mixture. The ANS intensity showed a 3.01 ± 0.12 fold increase (Table 1; fold increase was determined by the integration of the whole spectrum and all following values are referred to in the same manner) and λ_max_ was blue shifted from 520 to 480 nm in our experimental conditions. This observation is consistent with the fact that the Ca^2+^-bound CaM (Ca^2+^-CaM) exposes its hydrophobic surface, followed by ANS binding, in which the protein-bound ANS molecules are more shielded from the solvent. ANS intensity enhancements were also observed in all Ca^2+^-chimeras, with identical concentrations of the protein and dye, exhibiting increases in fluorescence ranging from 1.56 - 2.72 fold (Table 1). Our results appear to suggest that the chimeras have less hydrophobic surface exposure compared with CaM, consistent with the fact that there is no interaction between TnC and ANS [13]. However, such a conclusion does not provide instructive information because it is unclear on whether the ANS fluorescence enhancements were due to an increase in ANS lifetime (or quantum yield) and/or binding affinity. Thus, we turned to study CaM/chimera ANS binding by using isothermal titration calorimetry (ITC) and lifetime fluorescence.

**Table 1:** **ANS binding to CaM and chimeras by fluorescence and ITC**

	**ANS binding in the absence of salt**		**ANS binding in the presence of salt**		**ANS binding in the presence of salt and Orai** **-CMBD**	
	**30 mM Hepes, pH 7.5, 2 mM Ca** ^2+^		**30 mM Hepes, pH 7.5, 0.1 M NaCl, 2 mM Ca** ^2+^		**30 mM Hepes, pH 7.5, 0.1 M NaCl, 2 mM Ca** ^2+^	
**Protein**	**ANS enhancement (fold)** ^**a**^	**K**_app_ **(M** ^−1^ **)**	**ANS fold enhancement (fold)** ^**a**^	**K**_app_ **(M** ^−1^ **)**	**ANS fold enhancement (fold)** ^**a**^	**K**_app_ **(M** ^−1^ **)**
CaM	3.01 ± 0.12	2.10 ± 0.11 × 10^3^	3.49 ± 0.07	3.69 ± 0.08 × 10^3^	3.87 ± 0.10	3.43 ± 0.65 × 10^3^
CaM(1TnC)	2.72 ± 0.10	9.86 ± 1.24 × 10^2^	3.33 ± 0.06	1.13 ± 0.04 × 10^3^	3.31 ± 0.05	1.20 ± 0.08 × 10^3^
CaM(2TnC)	2.55 ± 0.05	1.01 ± 0.07 × 10^3^	3.01 ± 0.14	1.10 ± 0.01 × 10^3^	3.07 ± 0.04	9.70 ± 0.31 × 10^2^
CaM(3TnC)	1.56 ± 0.04	6.70 ± 0.51 × 10^2^	1.74 ± 0.08	8.56 ± 0.21 × 10^2^	2.24 ± 0.11	1.07 ± 0.10 × 10^3^
CaM(4TnC)	2.37 ± 0.22	1.80 ± 0.05 × 10^3^	2.90 ± 0.19	2.02 ± 0.03 × 10^3^	3.62 ± 0.15	2.67 ± 0.24 × 10^2^

^a^The ANS intensity enhancements were calculated from spectra integration and compared to the corresponding apo proteins in identical experimental conditions.

**Figure 3:**
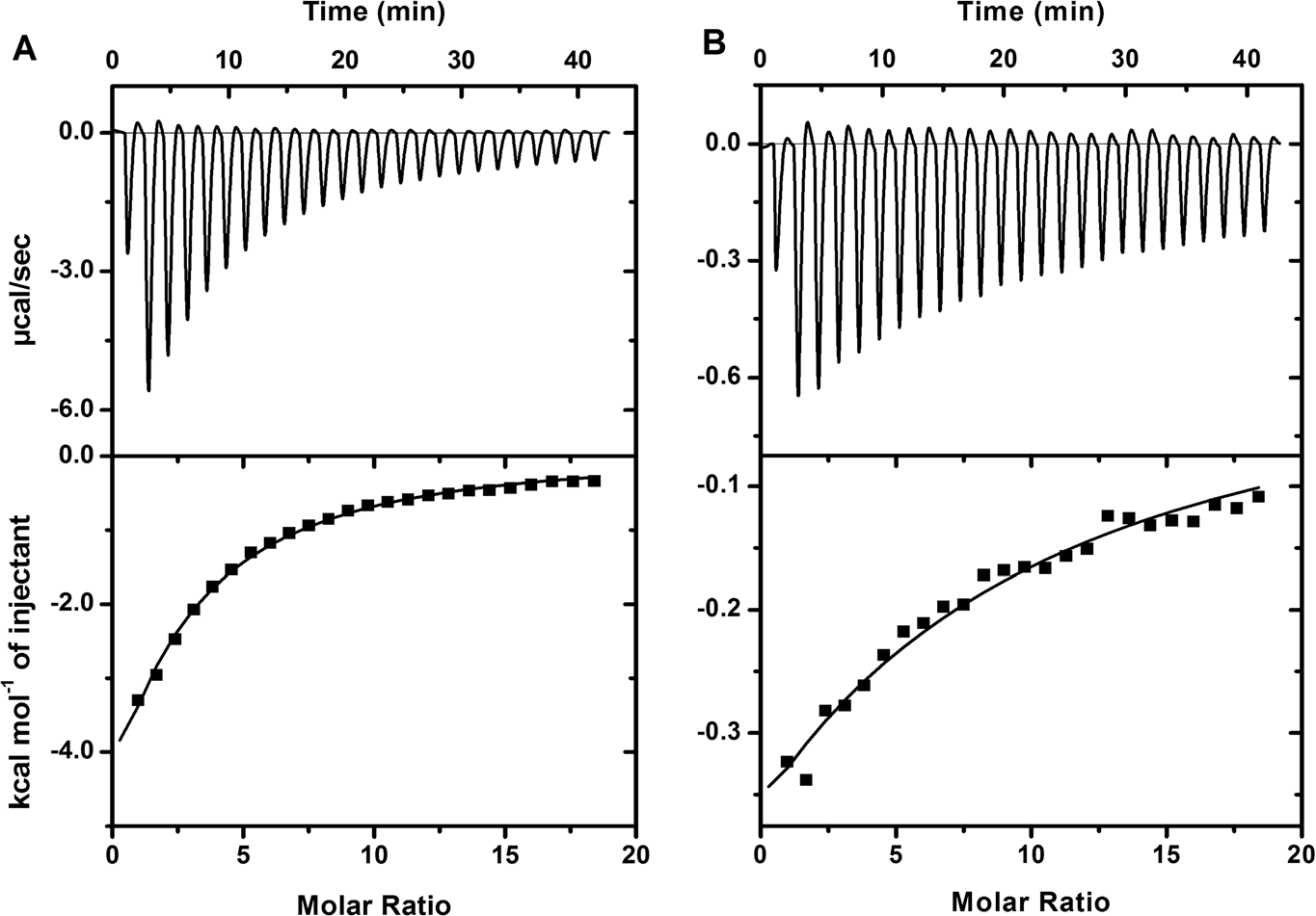
**ANS binding to CaM and CaM** **(3TnC)** **by isothermal titration calorimetry.** A solution of 50 μM CaM **(A)** or CaM(3TnC) **(B)** were titrated with 5 mM ANS at 25°C in the presence of Ca^2+^. The ITC data were fit with a “one set of sites” model as described in the Experimental section. The values from the fitting are shown in Table 1.

The raw ITC data for ANS binding to CaM is shown in Figure 3. The initial ANS injections resulted in heat released from the complex as shown in a downward heat rate. The heat evolved is proportional to the amount of the ANS/Ca^2+^-CaM complex formation. Apparently, the titration did not reach completion due to the weak ANS binding. We fit the data with “one set of sites” to obtain the apparent association binding constant (K_app_) of 2.10 ± 0.11 × 10^3^ M^−1^ for Ca^2+^-CaM (Table 1), consistent with a previous report [23]. Note that K_app_ is not the binding constant for the ANS molecule; rather it represents the overall low-affinity interaction of the multiple ANS molecules to CaM. It is unclear on how many ANS binding sites exist in CaM, given a range of 2–5 ANS molecules in CaM complexes have been reported [24,25]. Based on our studies, the middle point from the ITC thermogram (and the inflection point in the corresponding derived binding isotherm) indicates approximately 4–6 bound-ANS molecules in Ca^2+^-CaM. Therefore, K_app_ is proportional to the actual number of protein-bound ANS molecules in the experimental condition. In the text below, we will discuss K_app_ as the ANS binding affinity for simplicity. Almost all chimera proteins, except CaM(4TnC), showed a significant lower ANS binding affinity, which is parallel to the findings from fluorescence (Table 1). For example, CaM(3TnC) has the lowest ANS fluorescent enhancement (1.56 ± 0.04 fold) and binding constant (6.70 ± 0.51 × 10^2^ M^−1^). In the presence of 0.1 M NaCl, both the ANS fluorescence enhancements and association binding constants for CaM and chimera proteins increased (Table 1), indicating that the salt screens the repulsion between ANS and the acidic residues in the proteins. In the absence of Ca^2+^, no ANS binding was observed in the ITC experiments, consistent with the fluorescence studies.

**Figure 4:**
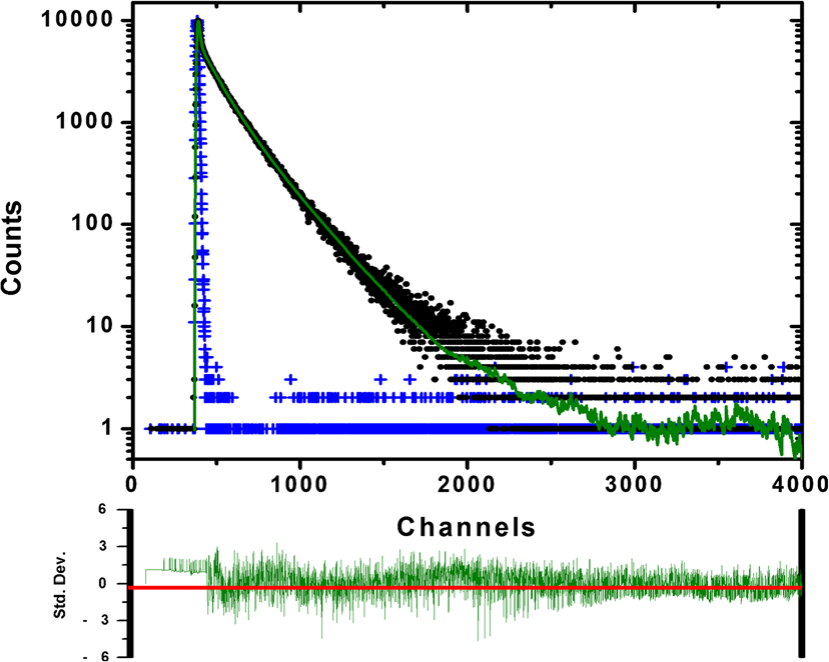
**The lifetime measurement of CaM-bound ANS.** A solution containing 5 μM CaM and 50 μM ANS was excited with 345 nm from a NanoLED light source and its dynamic fluorescence decay (black) was monitored with TCSPC using a 200-ns window for 4000 channels. The prompt is shown in blue color. The decay was best fitted with a three-exponential decay (green line in upper panel) and the weighted residues are indicated in the lower panel. The calculated χ^2^ is 0.986. The values from the fitting are shown in Table 2.

Given that steady state fluorescence cannot differentiate the intensity contribution from individual ANS molecules, we utilized time-correlated single-photon counting (TCSPC) spectrometry to measure the ANS fluorescence decay. A solution of 5 μM CaM and 50 μM ANS in the presence of 2 mM Ca^2+^ was excited with a 345 nm light source from a NanoLED and the emission was monitored with a 365 nm cutoff filter. The decay and the fitted function are shown in Figure 4. The data was best fit with a three-exponential-decay function. The shortest lifetime (τ_3_ = ~0.3 ns) was assigned to that of free ANS molecules [26], which was confirmed by using an ANS solution alone. Note that this lifetime reaches the detection limitation of our instrument and therefore, it cannot be determined precisely. Two longer lifetimes, τ_1_ = 6.4 and τ_2_ = 13.1 ns, were only observed in the presence of Ca^2+^, thus suggesting two different ANS populations with rather different probe environments. The fitting also provided the fractional (or relative amplitude), f_s_, which weights the “amount” of the emitting species, and the normalized pre-exponential value, B_s_, which provides the relative concentration of each species. The data for Ca^2+^-CaM indicates that the τ_1_ and τ_2_ species contribute more than 95% (i.e. f_1_ + f_2_) to the fluorescence intensity, but consist of less than 30% of the total species (i.e. B_1_ + B_2_). When decreasing the [ANS]/[CaM] ratio from 10 to 0.5, the fluorescence intensity is attributed to high “relative concentrations” of the two longer lifetimes, with f_1_ = 0.47, B_1_ = 0.33 and f_2_ = 0.49, B_2_ = 0.16. Double fluorescence lifetime of bound-ANS (or ANS heterogeneity) is commonly seen in proteins [27] and in molten globular structures [28], where the later showed that ANS has τ_1_ = ~2 ns and τ_2_ = ~6 ns, in which the short lifetime represents ANS molecules located on the protein surface, while the longer lifetime represents ANS in the protein matrix. The data shown here indicates that the CaM-bound ANS molecules are located in a very hydrophobic environment, limiting its solvent accessibility capacity. In fact, the Stern-Volmer quenching by acrylamide (see experimental section) revealed a linear decrease in two lifetime components, yielding K_SV_ values for the short and long lifetime (K_SV_(τ_1_) and K_SV_(τ_2_)) of 1.4 ± 0.2 M^−1^ and 0.25 ± 0.05 M^−1^, respectively (data not shown). The quenching rate constant (*k*_q_) for ANS with the short lifetime is 2.2 × 10^8^ M^−1^ s^−1^ while *k*_q_ for τ_2_ is 1.9 × 10^7^ M^−1^ s^−1^, indicating the later is less solvent accessible. In other words, the long lifetime component of ANS is one order less quenched by acrylamide.

It is unclear where the binding sites of the ANS molecules are located in CaM. Using the fragments from the tryptic cleavage of CaM, it has been suggested that two ANS molecules bind to the N- and C-terminal lobes [24]. However, the summation of ANS fluorescence from individual lobes is much less than that of the intact CaM. Therefore, we believe that there are multiple ANS binding sites for CaM, but only two types are distinguishable from each other based on the lifetime measurements. We expected that the interaction of chimeras and ANS might affect the lifetime and/or its population (thus contributing to different f values). Such information can not only be used to correlate with steady-state fluorescence, but also may provide information about the individual lobes.

The lifetimes of CaM(1TnC)- and CaM(2TnC)-bound ANS molecules are different to those of CaM, with a similar τ_1_ value but substantially larger τ_2_ value (~18 ns). The exchanged EFs in the N-terminal lobes appear to reflect a longer ANS lifetime. On the other hand, τ_1_ for CaM(3TnC) and CaM(4TnC) is smaller than that of CaM while their τ_2_ is similar to that of CaM, indicating the exchanged EF3 and EF4 have a less profound impact on the ANS short lifetime component. The average lifetime (<τ>) calculated from Eq. 2 for each chimera is displayed in Table 2, showing CaM(1TnC) and CaM2(TnC) have a significant longer ANS average lifetime (13–14 ns). Though a longer lifetime observed in CaM(1TnC) and CaM(2TnC) should attribute to a higher fluorescence intensity, such a contribution is shaded by the weaker binding as indicated by the ITC studies.

**Table 2:** **ANS lifetime** ^**a**^ **for the uncomplexed and complexed CaM and chimeras**

	**Without Orai** **-CMBD**					**With Orai-** **CMBD**				
	**Component 1**		**Component 2**		**Average lifetime** **(ns)**	**Component 1**		**Component 2**		**Average lifetime** **(ns)**
	**τ**_1_ **(ns)**	**f**_1_	**τ**_2_ **(ns)**	**f**_2_	**<τ>**	**τ**_1_ **(ns)**	**f**_1_	**τ**_2_ **(ns)**	**f**_2_	**<τ>**
Ca^2+^-CaM	6.4	0.44	13.1	0.53	9.0	6.8	0.20	16.9	0.66	13.1
Ca^2+^-CaM(1TnC)	6.2	0.21	17.8	0.70	14.4	6.3	0.31	17.3	0.57	12.7
Ca^2+^-CaM(2TnC)	5.9	0.20	17.7	0.68	13.7	5.8	0.18	17.3	0.71	12.9
Ca^2+^-CaM(3TnC)	4.4	0.29	12.1	0.51	7.4	4.3	0.30	16.2	0.57	11.7
Ca^2+^-CaM(4TnC)	5.1	0.19	12.9	0.70	8.6	6.0	0.16	17.1	0.72	13.5

^a^Data from the fitting are only shown as an average for clarity.

In summary, we observed a correlation between K_app_ values and fluorescence enhancements for ANS binding, which is on the order of CaM > CaM(1TnC) ~ = CaM(2TnC) > CaM(3TnC). The lower fluorescence enhancements observed in chimera proteins are dominated by a lower ANS binding affinity. The lifetime measurements also revealed ANS heterogeneity. The exchanged EFs appear to alter ANS lifetimes and fractional values (i.e. molecule population), with the exchanged EFs in the N-terminal lobe likely to increase the longer lifetime component while those in the C-terminal lobe are likely to slightly decrease the short lifetime component. CaM(4TnC) is the exception to the trend, with the second lowest in ANS fluorescence enhancement but the second highest ANS binding affinity. Here, the ANS fluorescence decrease due to the shorter lifetime (5.1 ns) is compensated by a higher ANS binding affinity (1.80 ± 0.05 × 10^3^ M^−1^). If ANS fluorescence of a protein truly reflects the hydrophobic area of the protein, then our data supports the previous reports that replacing CaM’s EFs with those of TnC results in a lower ability to expose hydrophobic surface area [13]. However, the extent of hydrophobicity determined from steady-state ANS fluorescence can be misleading, as it both over and underestimated ANS binding in the CaM/TnC system determined from ITC.

### Fluorescence studies reveal the Ca^2+^-dependent interaction between Orai peptide and CaM derivatives

We then studied how CaM and the chimeras interact with a synthetic 24 amino acid peptide corresponding to the CaM-binding domain of an Orai channel protein (Orai-CMBD). Since Orai-CMBD contains a Trp76 residue (numbering in Orai) and there are no Trp residues in CaM, Trp fluorescence can be used to investigate the interaction between Orai-CMBD and CaM. The excitation wavelength at 295 nm was chosen to eliminate the signal from CaM’s Tyr residues. The peptide alone exhibited low fluorescence with λ_max_ of 350 nm in a Ca^2+^-independent manner. Upon the addition of Ca^2+^-CaM, Trp fluorescence was enhanced 1.82 fold and λ_max_ was blue-shifted from 350 to 335 nm, suggesting that the Trp76 is embedded in a more hydrophobic environment provided by CaM. In an identical experiment but without Ca^2+^, no major signal change was observed upon the addition of CaM, supporting the conclusion that the interaction between Orai and CaM is Ca^2+^-dependent. The chimera proteins revealed a similar Trp fluorescence change with the trend of CaM > CaM(4TnC) = CaM(3TnC) > CaM(1TnC) > CaM (2TnC) (Table 3). The small difference in intensity change and λ_max_ suggests Trp76 is bound within the proteins in a similar manner.

**Table 3:** **Trp76 of Orai**-**CMBD fluorescence and Stern**-**Volmer quenching constants**

**Protein**	**λ**_max_ **(nm)**	**Trp fluorescence enhancement upon protein binding** **(fold)**	**K**_sv_ **(M** ^−1^ **) acrylamide**	**K**_sv_ ** (M** ^−1^ **) KI**
Orai-CMBD	350	n.a.^a^	13.5 ± 0.4	13.1 ± 0.5
CaM/Orai-CMBD	335	1.82 ± 0.06	1.35 ± 0.08	1.31 ± 0.08
(CaM)1TnC/Orai-CMBD	335	1.61 ± 0.05	1.80 ± 0.09	1.75 ± 0.06
(CaM)2TnC/Orai-CMBD	336	1.44 ± 0.11	1.95 ± 0.10	1.85 ± 0.05
(CaM)3TnC/Orai-CMBD	336	1.75 ± 0.08	1.32 ± 0.05	1.21 ± 0.09
(CaM)4TnC/Orai-CMBD	337	1.76 ± 0.07	1.51 ± 0.04	1.22 ± 0.10

^a^Not applicable.

The interaction of Orai-CMBD to Ca^2+^-CaM can also be monitored using ANS fluorescence. In the absence of Orai-CMBD, Ca^2+^-CaM and Ca^2+^-chimeras interact with ANS differently with λ_max_ = 480 nm for CaM, CaM(1TnC), and CaM(2TnC) and λ_max_ = 500 nm for CaM(3TnC) and CaM(4TnC) (Figure 2). The Orai-CMBD binding to CaM enhanced ANS fluorescence ~10%, mainly due to the resulting increases in lifetime (Table 2) but not affinity (Table 1). Orai-CMBD binding to CaM retains a similar short ANS lifetime (i.e. 6.4 versus 6.8 ns) but increases the long lifetime from 13.1 to 16.9 ns, respectively (Table 2), whereas Orai-CMBD binding to CaM(1TnC) and CaM(2TnC) neither increased ANS intensity enhancements, lifetime, and binding affinity. Orai-CMBD binding to CaM(3TnC) and CaM(4TnC) restored their λ_max_ from 500 to 480 nm and increased ANS intensities ~30% and 25%, respectively (Table 1). The fluorescence enhancement was primarily due to the increase in both lifetime and binding affinity. Our data revealed that exchanging an EF in the same lobe (N- or C-terminal) exhibits a similar structural effect probed by the ANS molecule, consistent with the functional dependency of a tandem EF pair in most EF-containing proteins.

The non-significant change of ANS lifetimes in the N-terminal ends of CaM(1TnC) and CaM(2TnC) suggests that peptide binding causes either minimal conformational change on that lobe or a structural change not sensitive to the ANS probe. On the other hand, CaM(3TnC) and CaM(4TnC) have a very similar response to that of CaM, suggesting the exchanged EFs in the C-terminal end retain their ability of conformational change for peptide binding.

### Thermodynamics and kinetics of Orai-CMBD to CaM and chimeras

**Figure 5:**
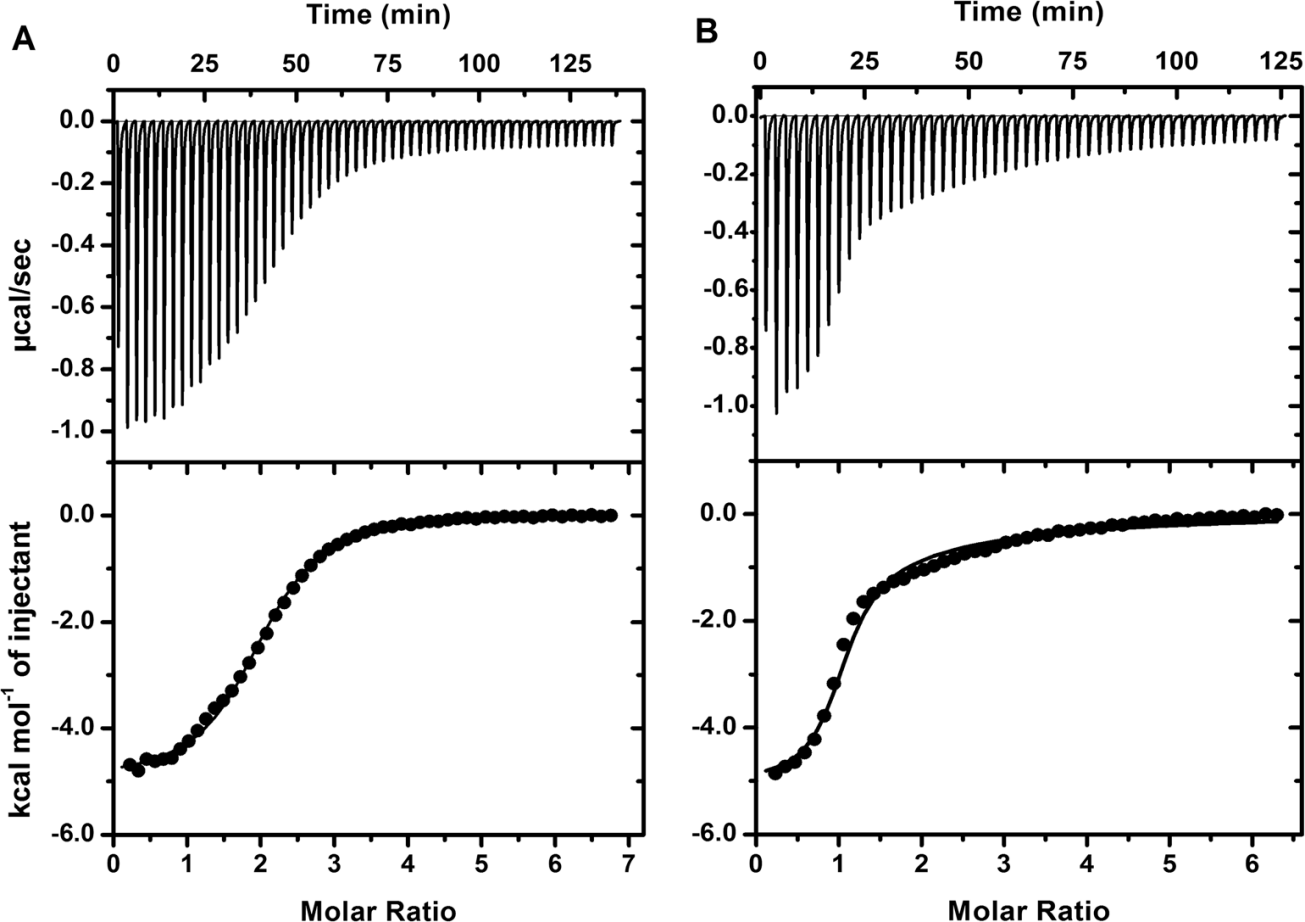
**The binding thermodynamics of Orai-CMBD to CaM and CaM(** **1TnC** **) determined by ITC.** A solution of 30 μM CaM **(A)** or CaM(1TnC) **(B)** was titrated with 1 mM Orai-CMBD in 10 mM Tris, pH 7.5, 0.1 M NaCl, 2 mM Ca^2+^. The upper panels show the heat evolved per injection and the lower panels show the integrated heat per injection versus the molar ratio. The model for the fitting was “one set of sites” for CaM and two sequential binding sites for CaM(1TnC). In both cases, the middle points of the thermograms indicates a stoichiometry of 1:2 protein:ligand binding. The values from the fitting are shown in Table 4.

The data from fluorescent studies indicated that the individual exchanged EFs alter the surface hydrophobicity but retain the binding to Orai-CMBD. However, it is unclear about the specifics of such an interaction. Thus, we used ITC to obtain the thermodynamics of Orai-CMBD to CaM and chimeras. The Orai-CMBD titration to CaM in the presence of Ca^2+^ at 25°C exhibited a typical calorimetric reaction (Figure 5A upper panel), in which the heat release per injection was observed. The heat evolved decreased gradually till the background signal was reached. The plot of heat evolved per injection (ΔQ_i_) versus molar ratio showed an exothermic, sigmoid-shape binding isotherm (Figure 5A lower panel). The ITC data was best fit into a “one set of sites” model and the K_a_ and ΔH values were determined to be 8.92 ± 1.03 × 10^5^ M^−1^ and −5.02 ± 0.13 kcal/mol, respectively, with N = ~2 (Table 4). The data agreed with the previous report [20] and the result indicates that CaM contains two Orai-CMBD binding sites, each with a similar binding affinity and enthalpy change that are not distinguishable with the calorimetric measurement.

**Table 4:** **Thermodynamics and kinetics of Orai**-**CMBD to CaM and chimeras**

**Protein**	**K**_a1_ **(M** ^**−1**^ **)**	Δ**H**_1_ **(kcal/** **mol)**	**K**_a2_ **(M** ^−1^ **)**	Δ**H**_2_ ** (kcal/** **mol)**	**Binding model or N for one set of sites**	***k***_off_ **(s** ^−1^ **)**
CaM	8.92 ± 1.03 × 10^5^	- 5.02 ± 0.13	n.a.^a^	n.a.	N = 2.05 ± 0.30	1.41 ± 0.08
CaM(1TnC)	7.57 ± 1.10 × 10^5^	- 4.99 ± 0.14	1.70 ± 0.30 × 10^4^	- 3.33 ± 0.30	Sequential	2.44 ± 0.21
CaM(2TnC)	6.66 ± 0.20 × 10^5^	- 5.21 ± 0.21	1.39 ± 0.35 × 10^4^	- 3.79 ± 0.39	Sequential	2.64 ± 0.32
CaM(3TnC)	2.90 ± 0.12 × 10^5^	- 6.58 ± 0.05	n.a.	n.a.	N = 1.93 ± 0.24	0.68 ± 0.25
CaM(4TnC)	5.77 ± 1.35 × 10^5^	- 5.34 ± 0.40	n.a.	n.a.	N = 1.87 ± 0.15	0.72 ± 0.31

^a^Not applicable.

The ITC measurements provide information about net non-covalent interactions, including hydrogen bonds, ionic interactions, and van der Waals (VDW) interactions, as well as water solvation. However, proton release or take-up from solvent in the formation of complex may contribute significantly to the apparent determined enthalpy (ΔH_app_). Therefore, the heat evolved due to buffer protonation/ionization has to be determined and corrected to obtain accurate binding information. Using different buffers, including HEPES, Tris, and POPS, we found no significant ΔH_app_ change (data not shown). Therefore, we concluded that there was no significant protonation in the CaM/Orai-CMBD complex, in which the obtained enthalpy difference becomes the binding enthalpy (ΔH_b_). We then performed similar experiments using the chimera proteins. The ITC raw data and thermogram of CaM(1TnC) and CaM(2TnC) clearly cannot be interpreted with a “one set of sites” binding model. It appears that the binding consists of a tighter binding with a higher ΔH_b_ followed by a weaker binding with a lower ΔH_b_ (Figure 5B). The ITC data of CaM(1TnC) can be best fit with a two sequential binding mode, yielding K_a1_ = 7.57 ± 1.10 × 10^5^ M^−1^, ΔH_b1_ = − 4.99 ± 0.14 kcal/mol and K_a2_ = 1.70 ± 0.30 × 10^4^ M^−1^, ΔH_b1_ = − 3.33 ± 0.30 kcal/mol. Because K_a1_ and ΔH_b1_ are close to that of CaM, those values are assigned to Orai-CMBD binding to the C-terminal lobe of CaM while the lower binding K_a2_ and the lower ΔH_b2_ are associated with the N-terminal lobe of the chimeras. This conclusion was further supported by the data from CaM(2TnC), which displayed a similar binding event as CaM(1TnC). Interestingly, the binding affinity and enthalpy change for Orai-CMBD complexed with CaM(3TnC) and CaM(4TnC) are comparable to those of CaM, suggesting that the impact from the exchanged EF3 and EF4 are less important for the binding. On the other hand, the exchanged EF1 and EF2 resulted in a one-order weaker binding affinity and a lower enthalpy change. Although it is a challenge to interpret the structural changes from the obtained thermodynamic parameters, our result still provides insights for the binding. The negative ΔH_b_ values presents the non-covalent bond energy for the complex formation while the positive entropy change, ΔS, reflects the entropic gain associated with desolvation, as seen in hydrophobic interactions. For CaM/Orai-CMBD, the driving force for the reaction is both enthalpic and entropic given the negative ΔH_b_ and positive ΔS (~10 cal/mol·K calculated from ΔG = ΔH – TΔS and ΔG = − RT ln(K_a_) where ΔG is the free energy change and T is temperature). Our data also suggested that the exchanged EF1 and EF2 in CaM(1TnC) and CaM(2TnC) either formed significantly lower non-covalent interactions and/or impaired the side chain packing and dynamics upon complex formation. It is very surprising to us that the hydrophobic surface probed by ANS does not correlate with Orai-CMBD binding given that CaM(3TnC) had the lowest ANS fluorescence but a comparable binding to Orai-CMBD. This contradicts the general concept that the hydrophobicity assessed by ANS reflects the ability of hydrophobic exposure.

**Figure 6:**
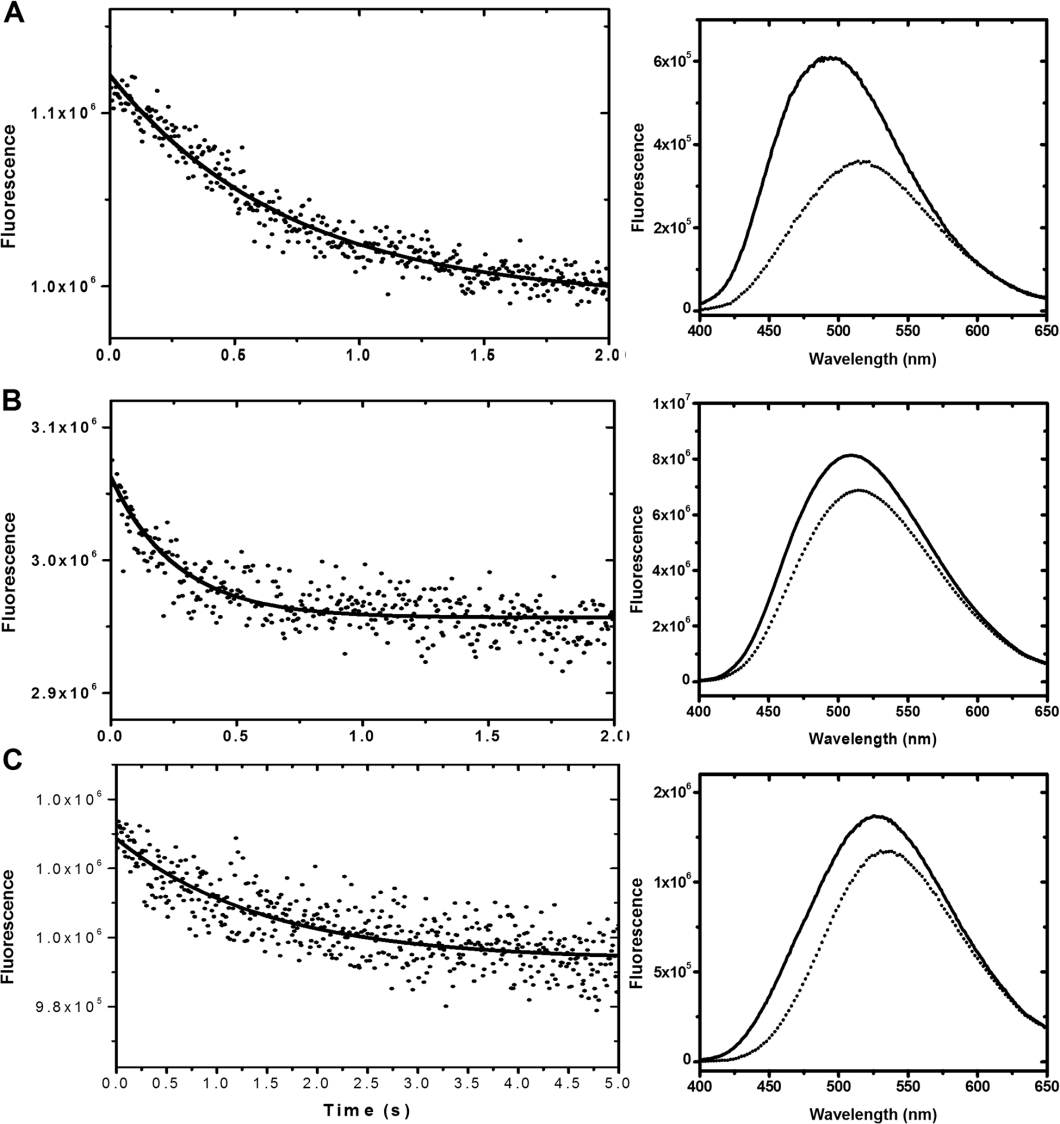
**Kinetics of Orai-CMBD to CaM and chimeras.** A solution of 5 μM dansyl-CaM, dansyl-CaM(1TnC), or dansyl-CaM(3TnC) and 15 μM Orai-CMBD was quickly mixed with a solution containing 75 μM CaM in the presence of 2 mM Ca^2+^ and the fluorescence decay was monitored for 2 s for CaM **(A)** and CaM(1TnC) **(B)** and 5 s for CaM(3TnC) **(C)** (left panel). The decay was fitted with a single exponential decay and the resulting *k*_*off*_ values are shown in Table 4. The peptide dissociated from dansyl-CaM or chimeras from the mixing resulted in the intensity decrease as observed in the complexed and uncomplexed fluorescently labeled CaM or chimeras (right panel).

To study the kinetics of Orai-CMBD associated or dissociated from CaM, we used a stopped-flow device to determine the association and dissociation rate constants, *k*_on_ and *k*_off_, respectively. The association was initiated by quickly mixing the Ca^2+^-CaM and Orai-CMBD solutions in the presence of Ca^2+^ and determined by its fluorescence, excited and monitored at 295 nm and 330 nm, respectively. Like other CaM/CMBD systems, *k*_on_ is too fast to be observed [29]. Orai-CMBD release from Ca^2+^-CaM was triggered by mixing a solution containing 5 μM dansyl-labeled CaM (dansyl-CaM) and an excess of Orai-CMBD with a solution containing 75 μM unlabeled CaM in the presence of Ca^2+^. The fluorescence of Ca^2+^-dansyl-CaM has a λ_max_ at 520 nm when excited at 370 nm. The Orai-CMBD binding resulted in a fluorescence increase and a λ_max_ blue shift to 490 nm. In the stopped-flow, the Orai-CMBD release from dansyl-CaM after mixing was promptly trapped by CaM, resulting in a dansyl fluorescence decrease (Figure 6A). The decay was best fit with a single exponential equation, giving *k*_off_ = 1.41 ± 0.08 s^−1^ (Table 4). Using the dansyl-labeled CaM(1TnC) and CaM(2TnC), the *k*_off_ values were determined to be 2.44 ± 0.21 and 2.64 ± 0.32 s^−1^, respectively (Figure 6B and Table 4). The peptide dissociation rates appear to be two-times slower for CaM(3TnC) and CaM(4TnC) (Figure 6C and Table 4). It is noteworthy to mention that only a single exponential decay was observed in all cases. Given that it is generally accepted that the two lobes of CaM function independently, we expected that either there would be two distinct *k*_off_ values exhibited for the chimeras if the kinetics are different for individual lobes, or a single *k*_off_ value (as seen in CaM) that reflects the Orai-CMBD dissociation from a specific lobe. It has been reported that the dansyl-labeling occurs in a single domain of CaM [30] and it is also suggested that the labeling site is near the N-terminal lobe [31]. If this is the case, then the obtained *k*_off_ values presented the Orai-CMBD dissociation kinetics specifically at the N-terminal lobe. This indicates that the lower ligand binding affinity (i.e. K_a2_) seen in the N-terminal lobe of CaM(1TnC) and CaM(2TnC) is partly due to the fast ligand dissociation rate. However, such an explanation cannot be applied to CaM(3TnC) and CaM(4TnC), in which the *k*_off_ values should be similar to that of CaM, if assuming an identical dansyl-labeled site. It is possible that our labeling in the exchanged EF3 and EF4 proteins exists within the other lobe given the very different dansyl fluorescence seen in CaM(3TnC) and CaM(4TnC). Thus, the slower ligand dissociation rate may be associated with the C-terminal lobe.

### Circular dichroism to monitor the secondary structure change upon Orai-CMBD binding

**Figure 7:**
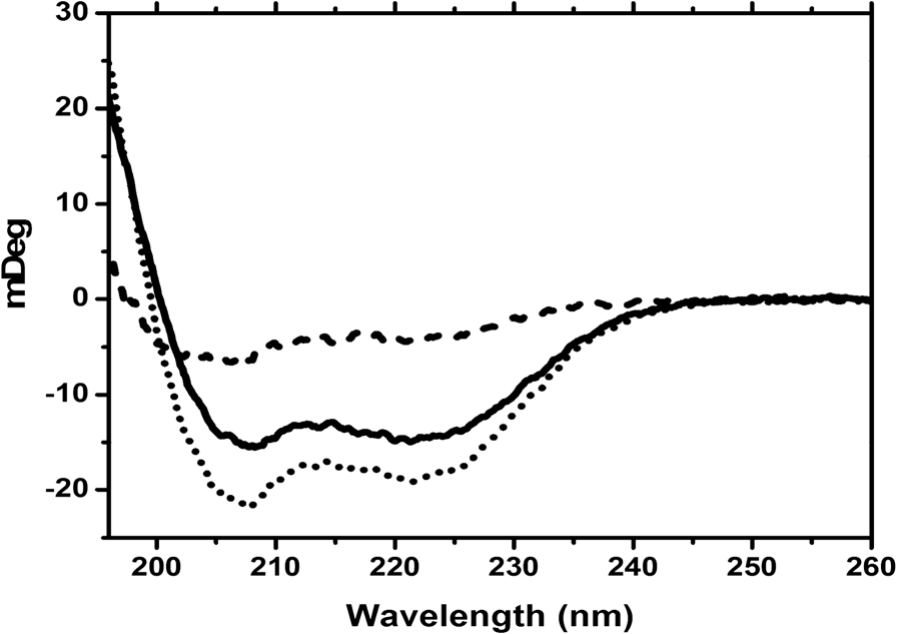
**Circular dichroism spectra of uncomplexed and complexed CaM.** The CD spectra of CaM were recorded in the presence of Ca^2+^ (solid line) and Ca^2+^ and Orai-CMBD (dotted line). Their differential spectrum (dashed line) presents the structure of Orai-CMBD in the complex assuming no CaM structural change upon ligand binding. Note that the CD unit was intentionally shown in degrees of light rotation because the CD spectra of CaM and the complexed Orai-CBMD have a very similar mean residual ellipticity.

We used circular dichroism (CD) to monitor the secondary structure of Ca^2+^**-**CaM upon Orai**-**CMBD binding. Ca^2+^**-**free CaM showed a shape typical of an α**-**helical structure with a negative peak (ellipticity or θ) at 220 nm and a more profound negative peak 209 nm (data not shown). In comparison to CaM, no significant difference was seen in all chimera proteins. Ca^2+^ binding induced a more α-helical formation in CaM and chimeras, which showed almost equal intensities at 220 and 209 nm. Because θ_220_ presents more closely to the α-helical content, whereas θ_290_ contributes more significantly from the β sheet and random coil structure, the ellipticity ratio at these two wavelengths (i.e. θ^220^/θ^209^) indicates the secondary structural change relative to the α-helix content. A perfect helix peptide or a protein with all helix structure has a θ_220_/θ_209_ value of 1.09. Ca^2+^**-**CaM contains α-helix content ranging from 45–60% in solution [32] and crystal structures [2]. The ellipticity ratio for Ca^2+^**-**CaM is ~ 1 due to the contained coil structure that exhibits a large negative ellipticity at 198 nm and slightly positive ellipticity at 205 nm. The CD spectrum of Ca^2+^**-**CaM/Orai**-**CMBD shifted to a more negative value at 220 and 209 nm, giving a θ_220_/θ_209_ value of 0.89 indicating significant structural changes for the complex formation. Given that there is no significant change observed in the uncomplexed and complexed CaM [8,9], the differential spectra (CD of the complex subtracted from that of Ca^2+^**-**CaM) indicates the secondary structure of Orai**-**CMBD in the complex (dashed line in Figure 7). The peptide shows a partial helix formation with θ_220_/θ_209_ = 0.71 consistent with the crystal structure. A similar observation was found for all chimeras, indicating secondary structure similarity among CaM and chimeras in the uncomplexed and complexed forms.

### Stern-Volmer quenching to determine the solvent accessibility of Trp76

**Figure 8:**
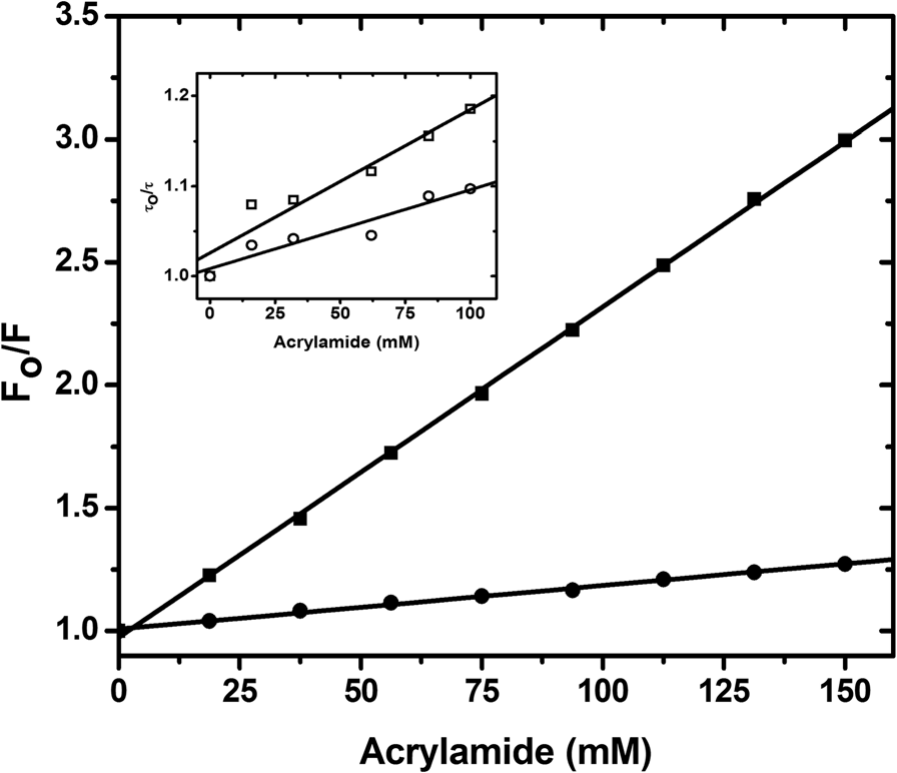
**Stern** **-Volmer plot of Trp76 fluorescence of Orai-** **CMBD quenched by acrylamide.** A representative figure of Stern-Volmer quenching by acrylamide is shown for Orai-CMBD and CaM/Orai-CMBD based on steady state fluorescence. To ensure no free Orai-CMBD peptide in the complex, CaM or the chimeras were added into the solution of 5 μM Orai-CMBD until no significant fluorescence change was observed. The values from the fitting are shown in Table 3. Insert: the quenching was monitored by two lifetime components of Trp76 of Orai-CMBD in the complex.

To investigate the microenvironment of Trp76 of Orai-CMBD, we employed collision quenching of Trp fluorescence by determining the accessibility of Trp residues to acrylamide and potassium iodide (KI) for the free and CaM-bound forms of Orai-CMBD. These two quencher molecules diffuse differently into the protein pocket and thus provide different environmental information for Trp. Fluorescence quenching is the result of Trp either being translocated to the surface of the protein, or Trp being located within the protein interior which allows acrylamide to diffuse. When Trp is being flanked by positively charged residues, the quenching becomes more effective with the use of an anionic quencher such as I^−^. In the employed conditions, the amounts of CaM and chimeras were added in excess (based on the molar ratio determined from ITC) to ensure that there was no free peptide in the solution. We only observed a linear quenching curve in Trp quenching at the concentrations used for acrylamide and KI (Figure 8). Orai-CMBD alone showed that its Trp residue was greatly accessible for quenching by acrylamide (K_sv_ = 13.5 ± 0.4 M^−1^) and KI (K_sv_ = 13.1 ± 0.5 M^−1^) (Table 3). It is not surprising that the Ca^2+^-CaM binding to Orai-CMBD rendered a better protection against Trp quenching with either acrylamide (K_sv_ = 1.35 ± 0.08 M^−1^) or KI (K_sv_ = 1.31 ± 0.08 M^−1^). The quenching studies were further extended by lifetime measurements. The fluorescence decay of Trp in Orai-CMBD, excited at 295 nm and monitored by a 325 nm cutoff filter, was best fit with a three-exponential-decay function with τ_1_ = 0.29, τ_2_ = 3.1 and τ_3_ = 7.5 ns, which are consistent to a complex lifetime observed for Trp in proteins. The addition of Ca^2+^-CaM to the Orai-CMBD solution increases the fractions, f_2_ and f_3_, for τ_2_ and τ_3_ significantly, but no information could be obtained about binding stoichiometry due to the complex Trp lifetime. Since the determination for τ_1_ is within the detection limitation of our TCSPC and its contribution to the overall fluorescence decay is negligible, we excluded it from quenching analysis. As shown in the insert of Figure 8, the τ_2_ and τ_3_ components both were quenched, but to a different extent. The K_sv_ for the longer lifetime τ_2_ (K_sv_(τ_2_)) is 1.52 M^−1^ and K_sv_(τ_3_) is 0.90 M^−1^. Since the steady-state K_sv_ is between the two K_sv_ values obtained from the lifetime measurement, it indicates that the quenching is a dynamic collision, not a static model. The lifetime quenching by KI exhibited similar results to those obtained by acrylamide.

Upon repeating similar studies using the chimera proteins, the Trp fluorescence in the complex with CaM(1TnC) and CaM(2TnC) was slightly more solvent accessible than CaM, while CaM(3TnC) and CaM(4TnC) displayed similar quenching values to those of CaM (Table 3). Interestingly, the Stern-Volmer values obtained from KI are very comparable to those from acrylamide studies, suggesting that the Trp76 of Orai-CMBD is embedded very deeply in a hydrophobic environment, such that the size and charged state of the quenchers cannot be used to differentiate the microenvironment. Our data aligns with the crystal structure, showing that Trp76 of Orai-CMBD is surrounded by several hydrophobic residues.

### Modeling and solvent-accessible calculations

To rationalize the experimental data on hydrophobic exposure upon Ca^2+^ binding, we modeled the chimera structures in the apo- and Ca^2+^-bound forms by aligning the sequences of chimeras to the models of apo-CaM (PDB: 1CFC) and Ca^2+^-CaM (PDB: 1CLL) as described in the experimental section, assuming that all chimera proteins adopt a similar structure to that of CaM. Modeling structures were then subjected to solvent accessible surface area (ASA) calculations for non-polar area (ASA_np_) and polar area (ASA_p_) using 1.4 Å as the van der Waals (VDW) radius and 5 for dot-density. The calculation agrees with conventional thinking in that the higher hydrophobic surface difference between apo and holo forms (i.e. ΔASA_ap_) is expected as seen in CaM, in which the apolar surface increases 555 Å^2^. However, a decrease of polar surface difference (ΔASA_p_), including those negatively charged residues, of 693 Å^2^ is also expected. Such surface changes are consistent with data from the ANS studies if one considers that ANS-sensitive proteins have to 1) induce more hydrophobic surface to interact with the aromatic portion of ANS, and 2) decrease the exposure of negatively charged residues so there becomes less repulsion between ANS sulfonate groups and the acidic protein. We then performed a similar calculation using the modeled chimera structures, in which the chimera backbone was aligned with CaM while the orientations of the side chains were optimized. Based on the calculation, there was no strong correlation between the surface exposure and experimental data as depicted in that of CaM(1TnC) and CaM(2TnC) having a more positive ΔASA_ap_ (733 and 699 Å^2^) and also a more negative ΔASA_p_ (−852 Å^2^ and −861 Å^2^). Nevertheless, the calculated ΔASA_ap_ areas (341 Å^2^ and 486 Å^2^) for CaM(3TnC) and CaM(4TnC) are consistent with the ANS binding from ITC and fluorescence studies, where the lower ANS fluorescence was observed. Note that the extra amino acids in CaM(1TnC) could not be modeled into the crystal structures and it was assumed that no conformational change upon Ca^2+^ binding occurred. Thus, the contribution of this extra sequence was canceled out in the ΔASA calculation. Similarly, the extra His-tagged sequence in 3TnC was treated in the same fashion.

Our experimental data suggests that chimera proteins such as CaM(1TnC) and CaM(2TnC) probably adopt a different structure than CaM in the N-terminal lobe. In fact, the structural alignment of CaM (PDB: 1CLL) and sTnC (4TnC) indicated that both structures cannot be aligned well given the root-mean square-deviation (RMSD) value of 6.8181 Å for all atoms. However, if a specific terminal lobe is aligned, the C-terminus gave RMSD = 0.856 Å while the N-terminus gave RMSD = 5.2 Å. Thus, it is unlikely that the N-terminal end of the chimera proteins, such as 1TnC and 2TnC, will adopt a similar structure as CaM.

The crystal structure of the CaM/Orai-CMBD complex is intriguing because the structure of Ca^2+^-CaM is not perturbed after ligand binding. The structural alignment of CaM complexed with Orai-CMBD to Ca^2+^-CaM reveals identical structures with RMSD = 0.459 Å. The experimental characterization in solution, including ours, all points to the complexation of two Orai-CMBD molecules that have similar binding affinities and occupy similar binding environments as judged from Trp76 fluorescence, but can still be differentiated by CaM(1TnC) and CaM(2TnC).

### Rationale of experimental data to the existing crystal structure of CaM/Orai-CMBD

The CaM/Orai-CMBD complex has been investigated by crystallography and NMR [20]. X-ray revealed that CaM adapts an unusual extended conformation with only one Orai-CMBD bound in the C-terminal lobe. However, NMR NOE indicates that CaM-N also interacts with Orai-CMBD, where both interactions for ligand binding are primarily hydrophobic. The reported ITC data also indicated a 1:2 stoichiometry of CaM/Orai-CMBD binding with the Orai-binding to CaM-C four times tighter (K_a_ = 9.1 × 10^5^ M^−1^) than CaM-N (K_a_ = 2.1 × 10^5^ M^−1^). Unambiguous evidence for the stoichiometry determination arose from the study of size exclusion chromatography, in which it showed that CaM is capable of binding two molecules of thioredoxin-fused Orai-CMBD. Therefore, the authors concluded that both the ligand binding sites of CaM-C and CaM-N are homologous. Our ITC data generally agreed with theirs and the fitting (“one site of sets” model and N = ~2) indicates that both binding sites are very similar in terms of thermodynamics. With the exchanged EF1 and EF2, the ITC thermograms showed a very interesting binding, which could only be fit with a two-sequential binding sites model. The results revealed that the N-terminal lobe of CaM(1TnC) and CaM(2TnC) have a 40 times lower binding affinity and a lower enthalpy change to Orai-CMBD than that of their C-terminal lobe. For a sequential binding, the ligand has to bind the higher affinity binding site before binding to the lower affinity site. The basis for this observation is not clear given that two lobes of CaM are considered to be functionally independent. Thus, our results possibly suggest that CaM(1TnC) and CaM(2TnC) have an unique structure, in which conformational change in domains may affect the central linker region that allows the signal to propagate from one lobe to the other. In TnC, the movement of helices that transmits the conformational change over substantial distances has been cited [33] and thermodynamic evidence supports the cooperativity of both lobes [34]. This might be particularly applicable to CaM(2TnC) since there are additional residues located in its linker region that are believed to alter the binding structure. On the other hand, the exchanged EF3 and EF4 have less profound impact on the Orai-CMBD binding, displaying a similar binding mode to that of CaM.

**Figure 9:**
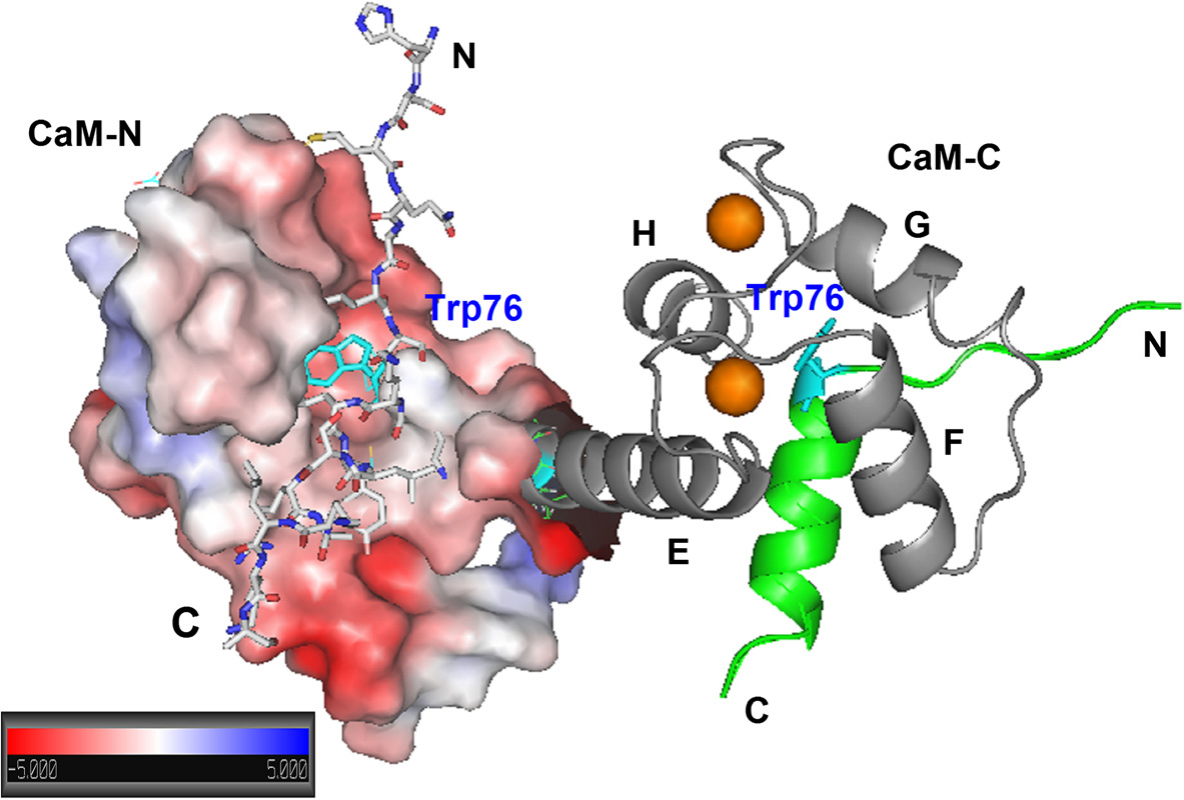
**The proposed model of the 1** **:2 CaM** **/Orai** **-CMBD complex.** The coordinates for the proposed model were obtained as a courtesy from Dr. Birnbaumer. One of two peptides (stick model) forms a partial helix structure and is bound in the hydrophobic patch (primarily gray) formed by CaM-N, shown as an electrostatic potential surface, where Trp76 (cyan) is deeply buried. The other Orai-CMBD shown in cartoon model (green) is flanked by the Ca^2+^-bound (orange) EF-hands in CaM-C.

**Figure 10:**
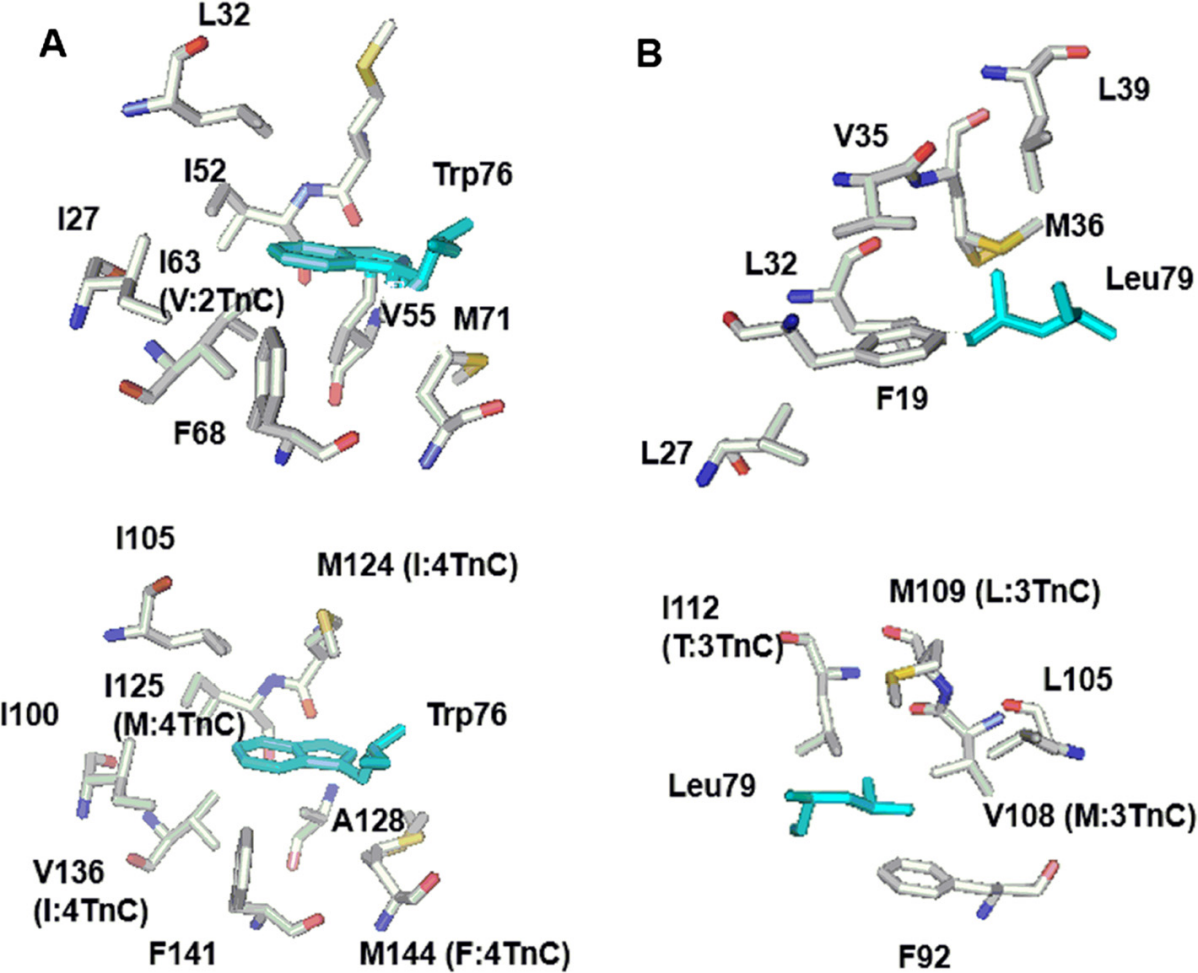
**The hypothetical interaction of Orai** **-CMBD to CaM-** **N and CaM** **-C.** The residues of Leu73, Trp76, and Leu79 are major contributors to the binding of CaM. In the Trp76 vicinity, the residues in one letter abbreviation in CaM-C are from the crystal structure while those in CaM-N are a hypothetical prediction **(A)**. The alteration in the position and chimeras are shown in parenthesis. Leu79 is located in the forming helix and interacts with hydrophobic residues **(B)**. Note that the numbering of residues is one residue off from Figure 1 since the first Met residue is not included in the crystal structure. We used the same numbering convention as the reference in this figure and within the text for clarity.

In the complex, Orai-CMBD adopts a partial helical structure in the crystal structure, which is confirmed in solution by our CD measurements. The Trp76 residue of Orai-CMBD is deeply buried in a hydrophobic patch formed by several hydrophobic residues in CaM-N and CaM-C (Figure 9). The peptide binds to a tunnel formed with a low-charged surface (gray color in the electrostatic potential map). The negatively-charged surface of CaM is primarily located near both ends of the peptide. Based on the crystal and NMR study, CaM/Orai-CMBD binding appears to be dominated by hydrophobic interactions from Leu73, Trp76, and Leu79 from Orai-CMBD with the hydrophobic residues provided from CaM. The electrostatic interaction formed by residues, such as Lys and Arg in the CMBD and Glu in CaM, as seen in other CaM/CMBD systems [1], is essentially not significant in this system. Those hydrophobic residues for the interaction in CaM are similar in the chimeras except for I63V (numbering in CaM) in CaM(2TnC) and M124I, I125M, V136I, and M144F in CaM(4TnC) (Figure 10A). The Met substitution to Ile, or vice versa, has been shown to have very minor effects on protein structure [35-37]. Similarly, other residue substitutions, such as Val and Phe, should retain the hydrophobic interaction with the ligand, given that the side chains of CaM are usually dynamic in solution [38]. Thus, it appears that the C-terminal lobe of the chimeras is flexible enough to adapt a structure similar to CaM to facilitate CMBD binding, as suggested by ANS lifetime fluorescence. On the other hand, the N-terminal lobe of the chimeras apparently has a different structure to that of CaM (even with only one residue change in the CMBD binding site of CaM(2TnC)) and/or is not flexible enough for packing its side chains for ligand binding, given the lower enthalpy and higher solvent accessible quenching as seen in ITC and quenching studies. Such results provide an explanation for the 40 times lower binding affinity than that of C-terminal lobe. Another key residue for the complex formation, Leu79, is also embedded into a hydrophobic surface in CaM-C, whereas CaM(3TnC) has additional residues substituted, such as V108M, M109L, and I112T (Figure 10B). The substitution of Ile with the polar residue Thr appears to have no major impact for ligand binding. However, the hypothetical model of CaM-N did not have optimal side chain packing for Leu79 and reorganization of its side chains is essential for binding. Nevertheless, the hydrophobic patches for interacting with Trp76 and Leu79 remain similar for CaM(3TnC) and CaM(4TnC), resulting in a compatible binding affinity. Apparently, the N-terminal lobe of CaM(1TnC) and CaM(2TnC) cannot be explained purely by residue substitution as depicted in modeling studies, suggesting they might adopt a dissimilar structure to that of CaM. The change in structure is most likely observed on the three dimensional level, rather than that of secondary structure given that CaM and chimeras have relatively similar extents of secondary structure in the apo and Ca^2+^ bound forms as demonstrated by CD.

## Conclusions

Here we used the fluorescent dye ANS to probe the structures of CaM and chimera proteins in the in the absence and presence of Ca^2+^. ANS studies revealed that the exchanged EFs alter ANS fluorescence intensity. However, such an intensity measurement, which is typically interpreted as “hydrophobicity”, should be carefully explained with the assistance of lifetime and binding affinity studies as described in this report. Lifetime measurements indicated ANS heterogeneity, in which two different ANS environments with distinct fluorescence dynamic decays were observed. Such ANS heterogeneity is most likely attributed to the two excited levels, which are altered by the exchanged EFs, especially EF1 and EF2. Among the chimeras, CaM(3TnC) appears to have a lower ANS binding affinity. Such a low induced hydrophobicity change might suggest an inability for CMBD binding. However, thermodynamics of CaM/Orai-CMBD revealed a poor correlation between ANS fluorescence and ligand binding, given that CaM(3TnC) still retains a similar binding affinity as that of CaM. Thus, our data strongly suggests that the induced hydrophobic surface assessed from ANS binding does not participate in the binding to Orai-CMBD. Rather, the determined hydrophobicity most likely reflects the interactions essential for enzyme activation, separate from CMBD binding. In fact, this conclusion agrees with the fact that Ca^2+^-CaM(3TnC) is not retained on hydrophobic interaction chromatography (HIC), unlike CaM and the other chimeras.

We also used CaM and chimera proteins to investigate the interaction with Orai-CMBD and rationalize our data assisted by a published crystal structure. Strictly speaking, our experimental data also can be explained by a canonical CaM/CMBD model given that only a single Orai-CMBD signal was detected in quenching and kinetic studies. However, the ITC data of chimeras clearly differentiates the two binding sites, thus a single peptide binding mode observed in some of our studies may be due to the homologous structures of CaM-C and CaM-N, such that average values were obtained. Our experimental data also indicates that the structural models for CaM(1TnC) and CaM(2TnC) are not reliable, especially in their N-terminal lobe. Additionally, it is unclear whether or not the exchanged EFs should have a similar impact on all CaM/CMBD systems because CaM in the CaM/Orai-CMBD complex adapts an unusual extended structure, interacting with the ligand primarily through hydrophobic forces. Our preliminary data from a collapsed 1:1 CaM/CaMKII model indicated a different observation in response to ANS (unpublished results). Its ANS fluorescence dropped 1.5 folds and shifted λ_max_ from 480 to 450 nm upon the complex formation. This decrease was not due to the change in ANS binding affinity, but rather the shorter lifetime component being significantly smaller compared with CaM/Orai-CMBD. Furthermore, one may also anticipate that the exchanged EF1 and EF2 will result in a similar impact on other CMBD binding. Our preliminary data has indicated in some cases the exchanged EF3 and EF4, not EF1 and EF2, impair the ligand binding. The study here addressed the first attempt to investigate the relationship of ANS-probed hydrophobicity and its interaction with a CMBD peptide via spectroscopy, thermodynamic and kinetic approaches. Thus, extensions from this study to other known CaM/CMBD systems and to the studies using a whole target enzyme will help understand the divergence of structure and function of CaM.

## Methods

### General

All chemicals were purchased from Sigma-Aldrich (St. Louis, MO) and Fisher Scientific (Pittsburg, PA), and were used without further purification. The peptide with the sequence corresponding to 68–91 residues of Orai1, _H2N_EHSMQALSWRKLYLSRAKLKASSR_COOH_, was purchased from NEO BioLab (Cambridge, MA). All characterization measurements were performed at least three times using three different protein batches, and the results were reproducible.

### Recombinant protein expression/purification

Chimera proteins, CaM(1TnC), CaM(2TnC), CaM(3TnC), and CaM(4TnC), were obtained as a generous gift from Dr. George [39]. CaM and the chimera proteins were expressed and purified as described previously [14]. In general, bacterial cells carrying the plasmid containing gene inserts of CaM(1TnC), CaM(2TnC), and CaM(4TnC) were induced for protein expression by heat shock at 42°C at an OD_600_ = ~1.0. The culture was continuously incubated for an additional 4–6 hrs at 42°C. CaM(3TnC) was sub-cloned to a pLW-His_6_ vector and was expressed as described previously for NOX5 [40]. All proteins except CaM(3TnC) were purified through a phenyl-sepharose column [41]. CaM(3TnC) was purified through a Ni-NTA column by elution with 40 mM imidazole. The purified proteins were stored at −80°C until further use. Protein purity was checked on SDS-PAGE electrophoresis and its purity was estimated to be > 95% based on density profiles measured using UN-SCAN-IT software (Silk Scientific, Inc., Utah). The concentrations of CaM and chimera proteins were determined as reported previously [20]. Dansylation of CaM and chimeras was performed in the presence of 2 mM Ca^2+^ as described previously [30].

### Isothermal titration calorimetry

The ITC experiments were carried out on a VP-ITC (GE, Pittsburgh, PA). Protein samples were buffer-exchanged to the desired buffer. Peptide concentration was prepared by adding buffer to the peptide powder as measured by weight. All samples were degassed 15–30 min before loading. To avoid buffer mismatch, the syringe and sample cells were rinsed with the desired buffer prior to sample loading. A typical titration was performed by sequential injections every 120–180 sec of 5–10 μL solution at 25°C. The ITC raw data was corrected for the heat of titrant dilution determined by an experiment conducted in identical conditions except that no protein was contained in the sample cell. The corrected data, after integration of heat evolved, was fitted with Origin software provided by the manufacturer to determine K_a_ and the observed or apparent ΔH (ΔH_app_). For ANS binding, a range of 5–50 μM CaM or chimera protein was titrated with 2–5 mM ANS solution in 30 mM HEPES, pH 7.5, 2 mM Ca^2+^, with or without 0.1 M NaCl. The Orai-CMBD binding to the proteins was determined by titrating a solution of 1 mM Orai-CMBD in 10 mM Tris, pH 7.5, 2 mM Ca^2+^, 0.1 M NaCl to 30 μM CaM or chimeras.

The possible buffer protonation/ionization to ΔH_app_ can be determined using different buffers with the known values of protonation enthalpy (ΔH_i_).

1 $$ \Delta {\mathrm{H}}_{\mathrm{app}}=\Delta {\mathrm{H}}_{\mathrm{b}} + \mathrm{n}\Delta {\mathrm{H}}_{\mathrm{i}} $$

where n is the stoichiometry indicating how many protons are released or absorbed to the buffer. A positive sign of ΔH_i_ indicates a protonation process while a negative denotes a deprotonation process.

### Spectroscopic measurements

Absorption measurements were carried out using a UV-1800 double-beam spectrometer (Shimadzu, Kyoto, Japan). The fluorescence spectra were recorded on a FluoroMax-3P (Horiba Scientific) equipped with a temperature control unit. For intrinsic Trp fluorescence, the excitation wavelength of 295 nm was chosen. Note that CaM and chimera proteins do not contain any Trp residues. The samples containing ANS or dansyl-labeled proteins were excited at 350 and 370 nm, respectively. The slit widths of 2 and 5 nm were typically chosen for excitation and emission, respectively, to eliminate photobleaching.

The fluorescence lifetime was measured with a TCSPC DeltaPro fluorometer (Horiba Scientific) at 20°C. For Trp lifetime measurements, a solution containing 15–20 μM Orai-CMBD with 5 μM protein was excited at 295 nm from a NanoLED295 source and the emission was detected with a 325 nm cutoff filter. For ANS lifetime measurements, each sample solution consisted of 5 μM CaM or chimera protein and 50 μM ANS in 30 mM Hepes, pH 7.5, 2 mM Ca^2+^ or 30 mM Hepes, pH 7.5, 2 mM Ca^2+^, 0.1 M NaCl. The lifetimes of free and bound-ANS were determined separately with the excitation at 345 nm from a NanoLED350 light source and the emission with a 365 nm cutoff filter. The decay distorted by the instrument response was corrected with a “Prompt” measurement using 0.01% Ludox AS40 colloidal silica (Sigma-Aldrich). The time windows for Trp and ANS measurements were 100 and 200 ns in 4000 channels with 10,000 peak counts, respectively. The dynamic fluorescence decay was fitted with a multiple-exponential decay function as shown in Eq. 2, with the DAS6 software provided with the instrument. Only those data with a chi square (χ^2^) value less than 1.2 were viewed as acceptable and reported.

2 $$ \mathrm{I}\left(\mathrm{t}\right) = \mathrm{A}+{\displaystyle \sum_{i=1}^n{\alpha}_i{e}^{-t/{\tau}_i}}\mathrm{and}\ {\mathrm{f}}_{\mathrm{i}} = {\alpha}_{\mathrm{i}}{\uptau}_1/{\displaystyle \sum_{i=1}^n{\alpha}_i{\tau}_i}\mathrm{and} < \uptau > ={\displaystyle \sum_{i=1}^n{\alpha}_i{\tau_i}^2}/{\displaystyle \sum_{i=1}^n{\alpha}_i{\tau}_i} $$

where I(t) is the fluorescence intensity at time t, α_i_ represents the pre-exponential factors, f_i_ is the fractional intensity, and < τ > is the amplitude-average lifetime.

The dissociation rate constant of the peptide from CaM or chimeras was determined by mixing a solution containing 5 μM dansyl-CaM (or dansyl-labeled chimeras), 15 μM peptide, 2 mM Ca^2+^ with a solution containing 75 μM CaM and 2 mM Ca^2+^. The solutions were excited at 370 nm and the signal changes at 497 nm (or 508 nm for CaM(1TnC) and CaM(2TnC) and 520 nm for CaM(3TnC) and CaM(4TnC)) were monitored. The integration and time intervals for all kinetic measurements were 2 and 5 ms, respectively. The fitting was carried out as described previously [41].

### Stern-Volmer quenching

The Trp fluorescence quenching of the Orai-CMBD peptide was carried out at 20°C by adding stock solutions of 6 M acrylamide and 1 M KI to a sample solution containing CaM and peptide in 50 mM Tris, pH 7.5, 2 mM Ca^2+^. The concentration of peptide was 5 μM and the concentrations of proteins ranged from 20 to 40 μM to ensure no free peptide in the solution. The emission intensity was recorded at 350 nm for peptide only and at 330 nm for the complex. The following Stern-Volmer equation was used for fitting:

3 $$ {\mathrm{F}}_{\mathrm{o}}/\mathrm{F} = 1 + {\mathrm{K}}_{\mathrm{sv}}\left[\mathrm{Q}\right] $$

where F and F_o_ are the fluorescence intensities at a given concentration of quencher ([Q]) and in the absence of quencher, respectively, and K_sv_ is the dynamic or collisional quenching constant.

For a quenching that is purely by collision, the steady-state and lifetime Stern-Volmer quenching equation can be expressed as

4 $$ <{\uptau}_{\mathrm{o}}>/<\uptau > = 1 + {\mathrm{K}}_{\mathrm{sv}}\left[\mathrm{Q}\right]\ \mathrm{and}\ {\mathrm{K}}_{\mathrm{sv}}={k}_{\mathrm{q}\ }\mathrm{x}\ {\uptau}_0 $$

where τ and τ_o_ are the lifetime in the presence and absence of the quencher, and *k*_q_ is the quenching rate constant.

### Far-UV circular dichroism (CD)

The CD spectra were recorded in a JASCO J-715 instrument (JASCO Corporation, Japan) equipped with a temperature control unit. CD spectra were recorded using a cylindrical 0.1 cm path length quartz curvet and were shown as the average of 3–6 scans using a spectra bandwidth of 1.0 nm. In all measurements, 0.1 mg/ml proteins in 10 mM Tris, pH 7.5 with 1–2 mM Ca^2+^ or 0.5 mM EDTA were scanned from 190 nm to 260 nm. Scans with the Orai-CMBD complexes were performed in identical conditions, with the addition of 10–12 μM peptide, calculated based on the molar ratio obtained from ITC studies.

### Modeling

The model structures of chimeras were generated and optimized with Swiss-Model Automatic Program Modeling Severs (http://swissmodel.expasy.org/) [42,43] using 1CFC and 1CLL as the templates for apo and holo CaM forms, respectively. The modeling program uses the information from experimentally determined protein structures to generate a model for a target protein (i.e. chimera in our case). Briefly, the template (i.e. crystal structure of CaM) was selected to perform target/template alignment and the optimization for all-atom models for the target sequence using ProMol-II [44] or MODELLER [45]. Finally, the model was subjected to a model qualifying evaluation assigned by the local scoring function QMEAN. GETAREA was used to calculate solvent accessible surface areas (http://curie.utmb.edu/getarea.html) [46]. The structures of Orai and chimera proteins were created in the same manner using the coordinates kindly provided by Dr. Liu *et al*. [20]. The electrostatic potentials were calculated using PyMol with Adaptive Poisson-Boltzmann Solver (APBS) plugin. First, a Poisson-Boltzmann calculation was performed using the PARSE force field in the PDB2PQR server (http://nbcr-222.ucsd.edu/pdb2pqr_1.8/) [47]. The resulting structure, containing charge and radius information, was used as an input for PyMol calculations. The following parameters were used for the calculation: 0.15 M for +1 and −1 ions, 310 K, 0.14 Å for solvent radius, and a dielectric constant of 2 and 78 for the protein and solvent, respectively.

## Abbreviations

ANS: 1-anilinonaphthalene 8-sulfonate
ASA: Accessible surface area
Ca^2+^: Calcium
CaM: Calmodulin
CaMKII: Calmodulin dependent kinase II
CD: Circular dichroism
CDI: Calcium-dependent inactivation
CMBD: Calmodulin-binding domain
EDTA: Ethylenediaminetetraacetic acid
ER: Endoplasmic reticulum
IPTG: Isopropyl-β-D-thiogalactopyranoside
ITC: Isothermal titration calorimetry
OD: Optical density
RMSD: Root-mean square-deviation
SOCE: Store-operated calcium entry
STIM: Stromal interaction molecule
TCSPC: Time-correlated single-photon counting
TnC: Troponin C
